# Mandarin Peels-Derived Carbon Dots: A Multifaceted Fluorescent Probe for Cu(II) Detection in Tap and Drinking Water Samples

**DOI:** 10.3390/nano14201666

**Published:** 2024-10-17

**Authors:** Marwa El-Azazy, Alaa AlReyashi, Khalid Al-Saad, Nessreen Al-Hashimi, Mohammad A. Al-Ghouti, Mohamed F. Shibl, Abdulrahman Alahzm, Ahmed S. El-Shafie

**Affiliations:** 1 Department of Chemistry and Earth Sciences, College of Arts and Sciences, Qatar University, Doha 2713, Qatar; aa1906650@student.qu.edu.qa (A.A.);; 2 Environmental Sciences Program, Department of Biological and Environmental Sciences, College of Arts and Sciences, Qatar University, Doha 2713, Qatar; mohammad.alghouti@qu.edu.qa; 3 Chemistry Department, Faculty of Science, Cairo University, Giza 12613, Egypt

**Keywords:** carbon dots, mandarin peels biochar, hydrothermal process, Plackett–Burman Design, fluorescence sensor, copper (II) detection

## Abstract

Carbon dots (CDs) derived from mandarin peel biochar (MBC) at different pyrolysis temperatures (200, 400, 600, and 800 °C) have been synthesized and characterized. This high-value transformation of waste materials into fluorescent nanoprobes for environmental monitoring represents a step forward towards a circular economy. In this itinerary, CDs produced via one-pot hydrothermal synthesis were utilized for the detection of copper (II) ions. The study looked at the spectroscopic features of biochar-derived CDs. The selectivity of CDs obtained from biochar following carbonization at 400 °C (MBC400-CDs towards various heavy metal ions resulted in considerable fluorescence quenching with copper (II) ions, showcasing their potential as selective detectors. Transmission electron microscopic (TEM) analysis validated the MBC-CDs’ consistent spherical shape, with a particle size of <3 nm. The Plackett–Burman Design (PBD) was used to study three elements that influence the F_0_/F ratio, with the best ratio obtained with a pH of 10, for 10 min, and an aqueous reaction medium. Cu (II) was detected over a dynamic range of 4.9–197.5 μM and limit of detection (LOD) of 0.01 μM. Validation testing proved the accuracy and precision for evaluating tap and mountain waters with great selectivity and no interference from coexisting metal ions.

## 1. Introduction

Carbon dots (CDs) are carbon-based materials of the nanoscale size range that have garnered attention in many scientific and technological fields. Thanks to their unique attributes, CDs hold potential for diverse applications: in electronics, as sensors, for bioimaging, drug delivery, and catalysis. Their unique quality is evident in their optical attributes, with significant photoluminescence yield, great sensitivity, and capability to operate in a concentration-dependent manner [1,2,3,4,5]. Therefore, CDs are often employed as sensing materials for numerous environmental pollutants, including, but not limited to, heavy metals [5,6,7,8]. Compared to the traditional molecular sensors, CDs are photostable, biocompatible and are readily surface-functionalized, which increases their solubility and allows for specific interactions with the target pollutant [9,10].

Recently, biomasses have gained a growing significance as carbon precursors for CDs due to their abundancy and the existence of heteroatoms, facilitating self-doping and resulting in excellent properties of the synthesized CDs [11,12,13,14]. One of the key benefits of using biomasses for environmental applications is their abundancy and lower toxicity, as compared to the synthetic carbon sources. Consequently, turning biomasses into CDs can boost the development and uses of CDs while lowering concerns of environmental pollution.

Additionally, biomasses can be converted into biochar via thermal treatment in an oxygen-deficient environment. The use of biochar as an adsorbent for a variety of environmental remediation applications has been widely reported and could be comprehended based on its unique attributes such as large surface area, carbon-rich structure, porosity, etc. [15,16,17]. On the other hand, applications entailing the use of biochar as carbon precursor for CDs are not as anticipated [18,19,20]. Several benefits could be garnered by using biochar as a precursor. To start with, and since biochar is made from biomass waste, it represents a sustainable approach that helps reduce total ecological impact via carbon fixation [21]. Furthermore, the synthesis of CDs on a large scale can be made economically feasible by using biochar, which is inexpensive and frequently derived from easily accessible resources [22]. The prevalence of functionalities like hydroxyl and carboxyl groups on the biochar surface facilitates the stabilization and surface functionalization of CDs [23]. On the other hand, synthesis of CDs using other carbon precursors might encounter some drawbacks, as illustrated in Table 1.

In this vein, several methods have been reported in the literature for the synthesis of CDs from their carbon precursors [5]. Yet, the hydro/solvothermal approach remains as one of the most reported routes, offering notable benefits in terms of cost-effectiveness, greenness, controllability, and simplicity [31,32].

Copper is recognized as a necessary micronutrient and a vital component for the survival of all living organisms, as it plays a crucial role in a variety of physiological processes [33,34,35]. Humans possess intrinsic mechanisms for regulating optimal copper levels within their bodies; however, surpassing the required limit can result in toxic symptoms such as vomiting, diarrhea, stomach aches, nausea, and a metallic taste. Also, excessive ingestion of copper ions, possibly from contaminated water sources, can cause a variety of health problems, affecting the liver or kidneys and causing gastrointestinal disorders [36]. The recommended daily copper consumption for healthy individuals is 900 μg/day, while newborns should consume up to 340 μg/day. Levels of copper present in the human body range from 1.4 to 2.1 mg/kg of body mass and are essential for sustaining a balanced metabolism. The US Environmental Protection Agency (EPA) has set a maximum limit copper level of 1.3 milligrams per liter [33].

By and large, several well-established analytical techniques have been reported in the literature for the determination of copper in different matrices with high sensitivity and selectivity. Commonly reported methods include the elemental analysis techniques such as inductively coupled plasma optical emission spectroscopy (ICP-OES), its mass spectrometry counterpart (ICP-MS), and atomic mass spectroscopy (AAS) [36,37]. ICP-MS, ICP-OES, and AAS are well-established analytical tools for copper analysis. ICP-MS is versatile, with low detection limits and multi-elemental analysis capabilities, but it requires sophisticated instrumentation and expert operators, making it costly to set up and operate [38]. While AAS is simpler and less expensive, it may lack sensitivity and multi-element capabilities, restricting its use to complicated matrices or trace-level analysis [39]. Sample preparation for these techniques can be time-consuming and involve the use of solvents and corrosive acids, raising safety concerns, while interference from matrix components can impair accuracy, demanding careful calibration [40]. Calibration standards and quality control methods are required for all techniques, which increases the overall analysis cost and time [41].

In the current approach, CDs derived from waste mandarin biochar has been employed as a sensitive and selective sensor for copper ions. Controlling the sensing process parameters while targeting the maximum fluorescence intensity (FI), and hence sensitivity, has been approached using Plackett–Burman design (PBD), offering multiple benefits, including less consumption of chemicals, less generation of waste, and implying a greener process [42,43]. The novelty of this work, therefore, stems from the facile, cost-effective, and ecofriendly synthesis of CDs from a natural and abundantly available waste, mandarin peels. This high-value transformation of waste materials into fluorescent nanoprobes for environmental monitoring represents a step forward towards a circular economy. Recycling of the waste materials into CDs offers a sustainable solution to waste disposal while creating a valuable, carbon-based nanosensor. The current approach not only reduces the negative impact of waste disposal, but it also provides a new pathway to produce CDs, with potential applications in the sensing of heavy metals. Additionally, it diminishes the demand for non-renewable resources.

A comprehensive spectroscopic characterization of the synthesized CDs has been approached. Furthermore, the selectivity of the prepared CDs towards copper (II) ions has been confirmed and compared to an array of other metal ions. The performance of the developed nanoprobes has been optimized, operating a parametric design control. Having developed these novel nano-probes, we have extended their application to real-world scenarios by testing their capability to detect the presence of copper in tap and mountain drinking waters. With a limit of detection as low as 0.01 µM, the high sensitivity is guaranteed in real samples where the copper (II) concentration is ~20 µM, as outlined by the US EPA. Comprehensive method validation has been fully depicted following the ICH guidelines.

## 2. Materials and Methods

### 2.1. Materials and Chemicals

Mandarin peels, which were bought from a local grocery store, were utilized to prepare the biochar (MBC). All the samples were dissolved in deionized water (DIW) acquired from the Millipore-Q system (Burlington, MA, USA). All chemicals were procured from Sigma-Aldrich (St. Louis, MO, USA). Quinine sulfate was utilized as a standard reference material to measure quantum yield (%QY). Iron (III) chloride anhydrous, tin (II) chloride, lead (II) acetate basic, cadmium (II) nitrate, chromium (III) chloride-6-hydrate, nickel (II) acetate tetrahydrate, and copper (II) acetate monohydrate were all used for the selectivity testing. Acetonitrile (ACN) and DIW were used as reaction media for the experimental design. Tap water was collected from laboratory facilities at Qatar University, and drinking water, which is imported from Lebanon with no label for the copper content on the bottle, was purchased from a Qatari supermarket. 

### 2.2. Instruments

A Teflon-lined stainless-steel autoclave was used to carry out the reaction under high temperature. An ultra-sonicator (SONYCLEAN PTY. Ltd., Adelaide, Australia) was utilized for proper mixing. The samples were filtered using regular filter paper and refiltered using a 0.45 μm syringe filter. A UV lamp (SPECTROLINE^®^, Model CM-10A, Melville, NY, USA) was used to observe the fluorescence. The excitation wavelength was determined using a UV–vis spectrophotometer (Agilent diode-array, Agilent, Santa Clara, CA, USA) with matched quartz cuvette cells. A Shimadzu (RF-6000, Kyoto, Japan) spectrofluorometer fitted with a 1 × 1 cm quartz fluorescent cuvette was used to perform fluorescence intensity (FI) measurements. The surface function groups of the produced CDs were analyzed using FT-IR spectroscopy (Perkin Elmer, Shelton, CT, USA). The morphology of the CDs was investigated using a Transmission electron microscope (TEM) (FEI Tecnai G2 S Twin FEG, Hillsboro, OR, USA) and Zen2.6 Lite software was used for calculating the particle size distribution. An X-ray diffractometer (X’Pert-Pro MPD, PANalytical Co., Almelo, The Netherlands) was used to perform the X-ray diffraction (XRD) investigation and a 2θ range of 5–90° was used for the measurements.

### 2.3. Preparation of Mandarin Peel Biochar (MBC)

The process involved removing the peels from mandarin fruit and rinsing them with tap water, followed by several washes with DIW. Subsequently, the peels were subjected to a temperature of 70 °C in an electric oven (Memmert, GmbH + Co. KG, Schwabach, Germany) for a duration of one week. The dried peels were turned into powder using a high-speed multi-function comminutor and then sieved through a 0.125 mm sieve to get rid of any large particles. The mandarin powder obtained was divided into four equal portions, where each portion was burnt at a specific temperature. Therefore, the 4 samples were placed into ceramic crucibles, compressed to eliminate any air bubbles, covered tightly, and heated in a furnace at different temperatures: 200 °C, 400 °C, 600 °C, and 800 °C, respectively, for 60 min. The resulting material was marked as (MBC) and was stored in centrifuge tubes.

### 2.4. Synthesis of the CDs

**Scheme 1:**
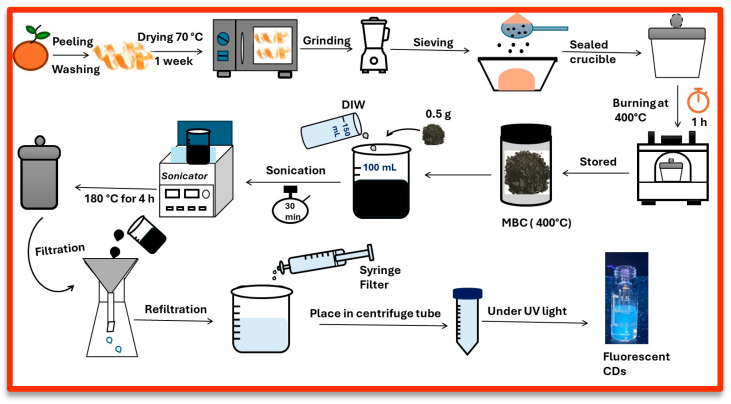
Synthesis of MBC400-CDs from waste mandarin peels.

Mandarin peel biochar MBC (0.5000 ± 0.0001 g) was dispersed in 150 mL of DIW. Then, sonication was carried out for a duration of 30 min. The solution was transferred into a Teflon-lined autoclave and placed inside the oven at 180 °C for 4 h. The autoclave was taken out and left to cool to room temperature. After cooling, the solution underwent filtration utilizing filter paper and re-filtered by 0.45 μm syringe filter. Finally, the solution was subjected to UV light to detect fluorescence. For FTIR and XRD analyses, materials were freeze-dried under vacuum for 48 h. An illustration of the synthesis procedure is revealed in Scheme 1.

### 2.5. Quantum Yield (QY) Measurements

Preliminary investigations included comparing the quantum yield (QY) of the CDs synthesized from the three biochars. Equation (1) was used to determine the QY, which was measured using 1.25 × 10^−3^ M of quinine sulfate dissolved in 0.1 M H_2_SO_4_.

(1) $$QY=Q_{r}\times \left(\frac{I}{I_{r}}\right)\times \left(\frac{A_{r}}{A}\right)\times \left(\frac{\eta^{2}}{\eta_{r^{2}}}\right)$$

In this equation, Q signifies the QY of the reference material, while *I* and *I_r_* stand for the integrated emission intensity areas of the CDs made for this investigation and the standard quinine sulphate, respectively. The UV absorbance of the CDs and the standard reference are denoted by the letters *A* and *A_r_*. Moreover, *η* and *η_r_* signify the standard reference and the CDs’ refraction index, correspondingly, where *η* = *η_r_* = 1.33.

### 2.6. Selectivity Testing

Solutions of CDs were scanned at various excitation wavelengths (λ_ex_), and the emission wavelength (λ_em_) was detected between 320 and 500 nm. The greatest FI was achieved at λ_ex_ = 310 nm and λ_em_ = 410 nm. The synthesized CDs were tested for their selectivity towards various metal ions, including iron (III), tin (II), lead (II), cadmium, chromium (III), nickel (II), and copper (II). For this purpose, stock (50-ppm) solutions of each tested material were prepared in DIW. Typically, 500.0 µL of the CD solution (MBC400-CDs) was added into the fluorescence cuvette, followed by 500.0 µL of buffer solution with a pH of (9.00 ± 0.02). 2.0 mL of DIW was the n added, followed by 500.0 µL of the analyte stock solution as well. Solution was thoroughly mixed, and the FI was measured. The ability of the tested materials to reduce FI was used to evaluate the selectivity of the synthesized CDs. The fluorescence quenching was determined by the ratio F_0_/F, where F_0_ represents the FI of the CDs, and F represents FI at 410 nm in the presence of the tested materials. All readings were compared against a blank sample containing all reaction components except the analyte.

### 2.7. Plackett–Burman Design (PBD) Copper (II) Detection

PBD was employed to find the optimum conditions for copper (II) detection, with the aim of achieving maximum sensitivity (highest fluorescence quenching, F_0_/F). The design was generated using Minitab 20 software (State College, PA, USA). PBD is characterized by a first-order polynomial mathematical model, depicted in Equation (2) [42,43,44,45].

Y = β_0_ + Σβ_*i*_
*x*_*i*_ (2)

In this equation, Y represents the response variable, F_0_/F, while β_0_ denotes the mean intercept, *x_i_* stands for the independent variable influencing the response, and β*_i_* represents the associated coefficient. Three factors were examined: contact time (CT), pH, and reaction medium (RM). Table 2 outlines these factors, along with their respective levels. The experimental design comprised 36 base runs conducted with 1 replicate each, totaling 36 runs, distributed across 1 block. Additionally, 12 central points were included in the design scenario. A stock solution of 50 ppm copper (II) was prepared in DIW, and to illustrate the experimental setting, 500.0 µL of CDs was added to a 3.5 mL cuvette, followed by 500.0 µL of buffer solution (with pH determined according to Table 3), 2.0 mL of the reaction medium (either DIW or ACN), and 500.0 µL of the copper (II) stock solution. The reaction mixture was allowed to incubate for a duration ranging from 30 s to 10 min, as outlined in Table 3. All the measurements were conducted in triplicate, and in all cases, measurements were compared against reagent blanks prepared in a similar manner but excluding copper (II).

### 2.8. Detection of Copper (II) in Real Samples

The content of copper (II) in both tap and drinking water was determined using the standard addition method. The technique included adding 500.0 µL of CDs, 500.0 µL of buffer (pH = 10.0 ± 0.2), 2 mL of DIW, and 200 µL tap water. Varied quantities of 50-ppm copper (II) standard solution were added to achieve final copper (II) concentrations ranging from 5.21 to 25.4 µM. The FI was measured using λ_ex_ of 310 nm, and the fluorescence emission intensity was measured at λ_em_ of 410 nm. The same procedure was repeated for the drinking water, and recovery calculations were executed in both cases using the ‘taken’ and ‘found’ concentrations of copper (II).

## 3. Results and Discussion

### 3.1. Optical and Structural Characterization of the Prepared CDs

#### 3.1.1. UV–Vis Spectrophotometry

**Figure 1:**
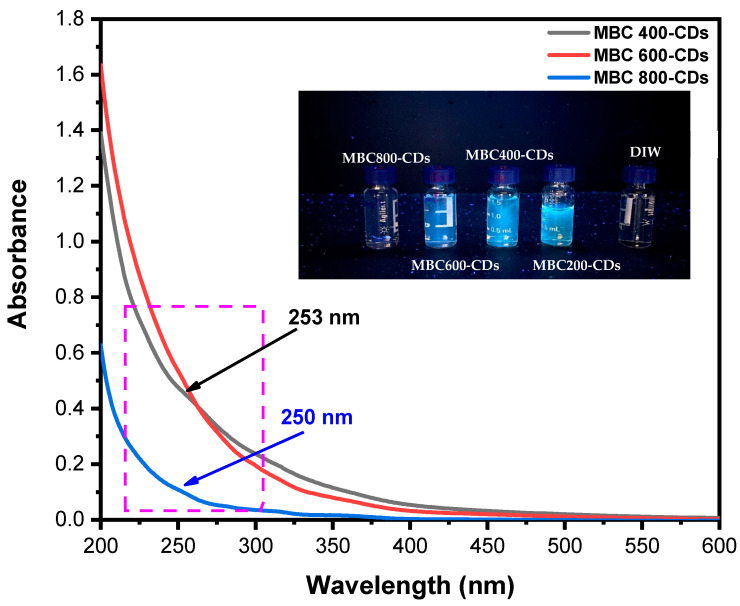
UV–vis spectra of the as-prepared MBC400, 600, and 800-CDs, including an inset image showing the CDs samples under UV light at 365 nm compared to DIW (far right).

The synthesized CDs were successfully dispersed in water, yielding a transparent solution. Under the UV light at 365 nm, the as-synthesized CDs exhibited a bluish fluorescence emission, as shown in the inset (Figure 1). The produced CDs solutions were analyzed using UV–vis spectra, as depicted in Figure 1. The three MBC400, 600, and 800-CDs spectra absorb in the UV region, with a weak shoulder peak between 220 and 255 nm (at 253 nm for MBC400-CDs). This shoulder may be ascribed to the π–π* transitions of the aromatic C–C and C = C bonds generated from the aromatic biochar π-system [46,47,48]. No absorption hump could be noted near 340 nm, implying the presence of a relatively broad size distribution of CDs [46].

**Figure 2:**
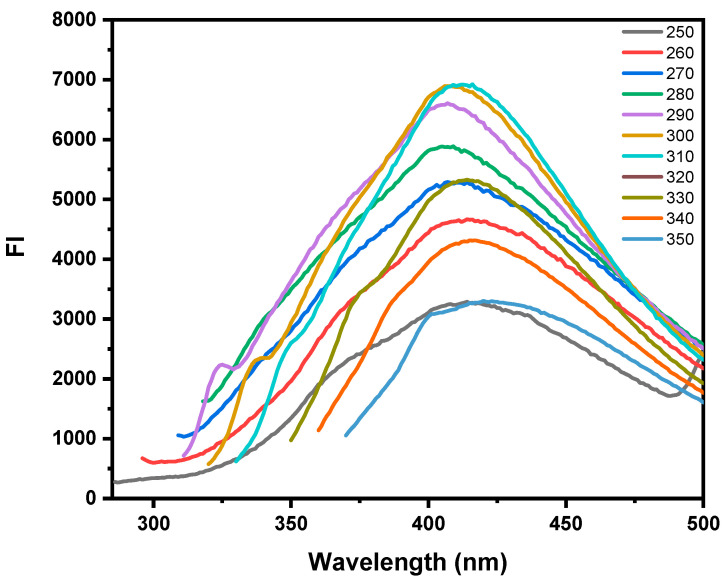
Fluorescence emission spectra of the as-synthesized MBC400-CDs emitted using different excitation wavelengths in the range between 250 and 350 nm.

Figure 2 shows that MBC400-CDs featured excitation-dependent fluorescence emission characteristics, allowing for wavelength adjustable emission. The red shift in emission intensity happened when the excitation wavelength was altered from 330 nm to 350 nm. The CDs were best stimulated at 310 nm, yielding the highest emission intensity. The maximum photoluminescence intensity of MBC400-CDs is centered at 410 nm.

#### 3.1.2. Selection of the Carbon Precursor

The carbon precursor for CDs synthesis was selected after testing four biochar samples: MBC200, MBC400, MBC600, and MBC800. The CDs generated from these biochars were principally compared in terms of their quantum yield (QY%), as shown in Table 4. Among these, the MBC400-CDs showed the highest QY%, reaching 7.31%. This finding conforms with earlier research suggesting that biochar produced below 400 °C might not be fully carbonized [49]. For this reason, the MBC200-CDs were difficult to filter. Furthermore, the data indicated that as the pyrolysis temperature rose from 400 °C to 800 °C, the QY% decreased. This trend is consistent with previous studies that observed a decline in photoluminescence with higher carbonization temperatures [46]. Therefore, the MBC400-CDs sample was chosen for further investigation. A comparison between the QY% of different CDs prepared from biochar as a precursor is reported in Table 4. The comparison shows that the current CDs derived from MBC possess a quite similar QY% when matched to the reported biochar-derived CDs, as shown in Table 5.

#### 3.1.3. Transmission Electron Microscopy (TEM)

**Figure 3:**
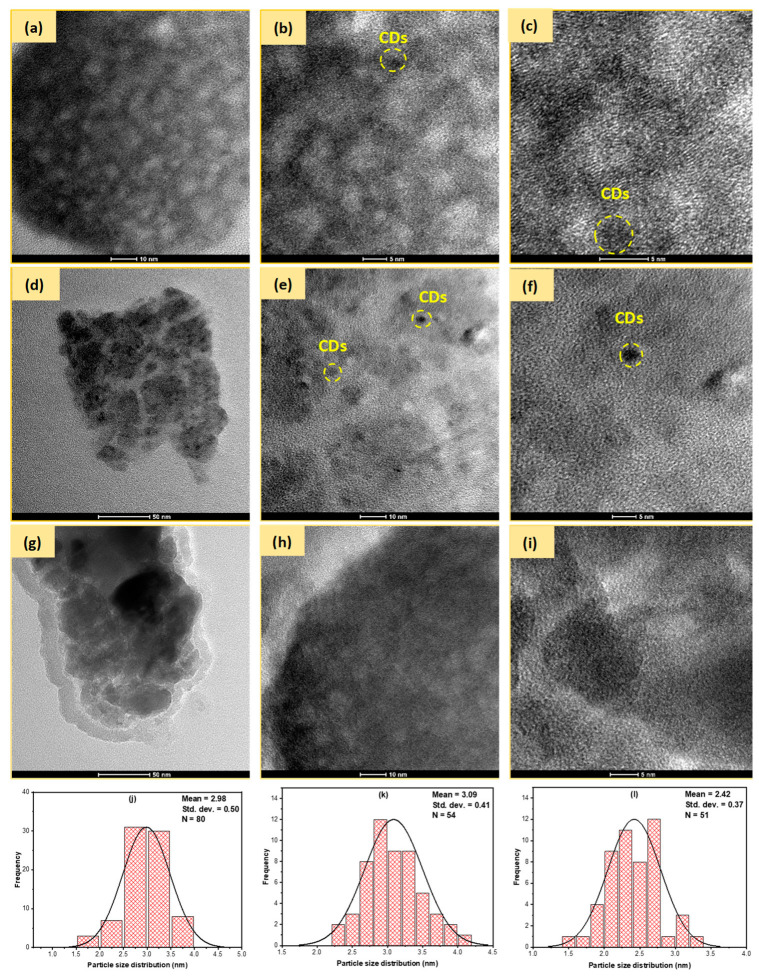
TEM micrographs of the prepared samples: (**a**–**c**) MBC400-CDs, (**d**–**f**) MBC600-CDs, and (**g**–**i**) MBC800-CDs at different scales between 5 and 50 nm. Micrographs denoted by the letters (**j**–**l**) are the PSD of the prepared samples from MBC400, 600, and 800, respectively.

TEM pictures revealed the morphologies and particle sizes of the as-prepared CDs. As shown in Figure 3a–i, most particles possess a uniform spherical shape. The particle size distribution (PSD) (Figure 3j–l) shows that MBC400-CDs have a particle size of 2.98 ± 0.50 nm, the PSD of the MBC600-CDs is 3.09 ± 0.41 nm, and the MBC800-CDs had an average size of 2.42 ± 0.37 nm. The MBC600-CDs sample has the largest PSD followed by MBC400-CDs, and MBC800-CDs sample has the smallest average size. The inset in Figure 3c shows the presence of tiny CDs with an inner core of 0.22 nm and an outer shell with amorphous structure which is characteristic for the CDs [52,53].

#### 3.1.4. Fourier Transform Infrared (FTIR) Spectroscopic Analysis

**Figure 4:**
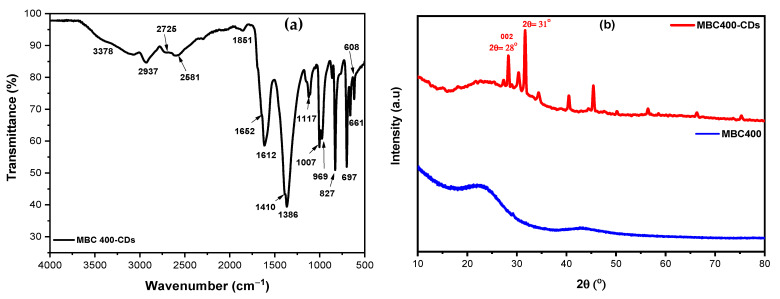
(**a**) FTIR spectrum of MBC400-CDs and (**b**) powder XRD pattern of the samples MBC400 (blue line) and MBC400-CDs (red line).

FTIR spectra were employed to explore the functional groups on the surface of the produced MBC400-CDs, Figure 4a. The FTIR absorption spectra showed a broad absorption band at 3378 cm^−1^, revealing the presence of O–H stretching vibrations mode. The broad band could be attributed to the presence of water with acidic functional groups. The two absorption bands at 2937 and 2725 cm^−1^ revealed the presence of CH_2_ antisymmetric stretching vibrations and CH_2_ symmetric stretching vibrations, respectively [54]. A significant absorption band at 1612 cm^−1^ indicates C=C stretching vibrations, confirming the presence of *sp*^2^ structures in the produced CDs [55,56,57]. Moreover, the peak at 1851 cm^−1^ as well as at 1652 cm^−1^ could indicate the presence of C=O stretching vibrations [58]. In contrast, a strong peak at 1386 cm^−1^ could be ascribed to C–N stretching vibrations. The two peaks at 1117 cm^−1^ and 1007 cm^−1^ could be associated with aromatic C–H in-plane bending vibration. On the other hand, the absorption bands in the range between 969 and 661 cm^−1^ show the presence of aromatic C–H out-plane bending vibrations [59,60]. The peak at 608 cm^−1^ could be attributed to the vibrations originating from the aromatic carbon [61]. The obtained data confirm the aromaticity of the formed CDs with the presence of important functional groups, including C=O and C–N, which positively affect the reaction capability towards metal ions.

#### 3.1.5. X-ray Diffraction (XRD)

The structural properties of both MBC400 and MBC400-CDs were analyzed using the X-ray Diffraction pattern (XRD) depicted in Figure 4b. The XRD pattern of MBC400 (blue line) shows the presence of a broad peak in the range between 2θ: 17° and 32°, confirming the presence of an amorphous structure of the MBC400 [62]. On the other hand, the XRD pattern of the prepared CDs (red line) shows the presence of some significant peaks compared to the parent biochar sample, including a broad and intense diffraction peak at 2θ = 28 and 31°, corresponding to the (002) plane of carbonaceous CDs. This peak aligns with earlier structural analyses of carbon quantum dots, as documented in JCPDS Card No. 26–1076 [63,64].

#### 3.1.6. Stability Testing of MBC400-CDs

**Figure 5:**
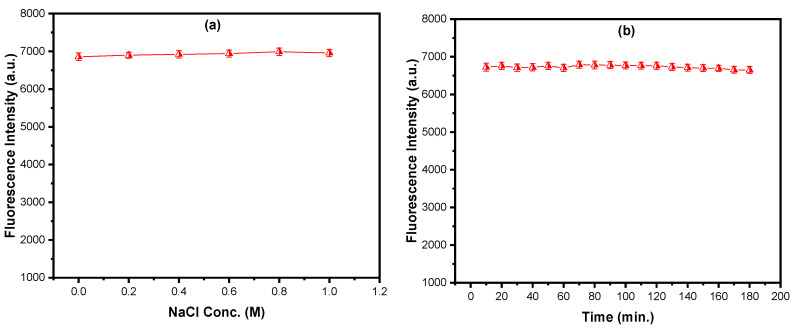
(**a**) The MBC400-CDs fluorescence intensity (FI) measured in different concentrations of NaCl and (**b**) MBC400-CDs FI measured versus time.

The stability of the MBC400-CDs fluorescence was investigated by recording the response, FI, at different concentrations (0.2–1.0 M) of NaCl. The obtained data shown in Figure 5a reveal no changes in the fluorescence intensity of the MBC400-CDs, even when the NaCl concentration reached 1.0 M, confirming the remarkable stability of CDs under high ionic strength conditions. The photostability of the MBC400-CDs was also studied by noting the change in FI over 3 h (continuous irradiation). The collected data shown in Figure 5b reflect the high stability of MBC400-CDs over a long period of time. Both plots confirm the stability of MBC400-CDs, with no significant decrease in the fluorescence intensity.

### 3.2. Selectivity Analysis

**Figure 6:**
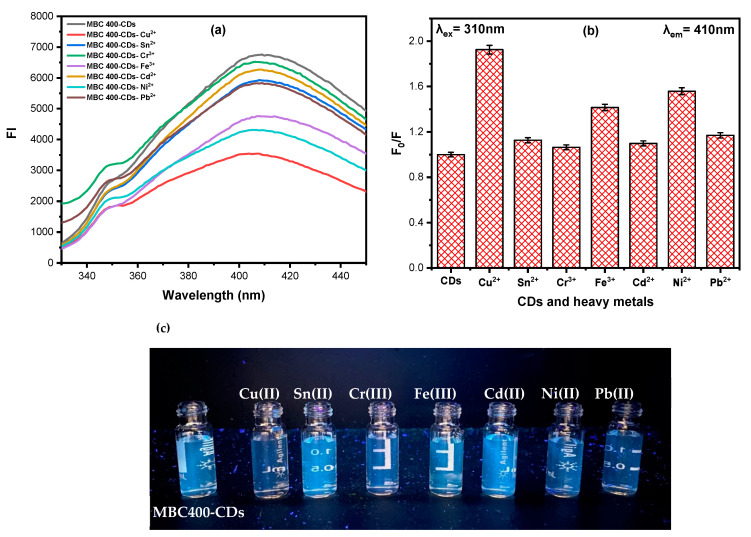
(**a**,**b**) is the selectivity test of the prepared MBC 400-CDs towards different metal ions, (**c**) a photo showing the MBC400-CDs sample before and after quenching using different heavy metal ions under irradiation using a longer wavelength UV lamp.

Figure 6a shows that the addition of various metal ions reduced the FI of MBC400-CDs to different levels. Figure 6b confirmed the quenching potential of each metal, where tin (II), chromium (III), cadmium (II) and lead (II) generated a minor quenching effect. On the other hand, iron (III) and nickel (II) produced a more noticeable effect. Copper (II) caused a significant drop in the FI, reaching a quenching percentage of 48.1% under non-optimum conditions.

Moreover, the interference of different heavy metals with copper (II) detection using MBC400-CDs was evaluated by determining the tolerance limit towards ions that caused quenching of the fluorescence of MBC400-CDs but to a lower extent compared to copper (II) ions. Tested ions included iron (III), cadmium (II), nickel (II), and chromium (III). The tolerance limit is the concentration of interfering ions that causes a deviation in the copper (II) quenching effect ≥ 10% [65]. For this purpose, the quenching effect of 25 µM copper (II) was measured first, followed by the addition of varying concentrations of the interfering ions, ranging from 5 to 200 µM. The tolerance limit was then calculated using Equation (3) as follows:

(3) $$Tolerance\;limit=\;\left|\frac{F_{0}-F}{F_{0}}\right|\times 100$$

In this formula, *F*_0_ is the fluorescence intensity of MBC400-CDs in the presence of 25 µM copper (II) solution measured at the optimum conditions, and *F* is the fluorescence intensity after the addition of the interferent ion. The obtained data shown in Table 6 reveal that the tolerance limit was lower than 10% for iron (III) (1.05–7.41%), chromium (III) (0.21–3.14%), and cadmium (II) (0.17–3.04%) at all tested concentrations, implying that the existence of these interferent ions has a slight effect on the detection of copper (II) using MBC400-CDs. In contrast, nickel (II) shows a higher deviation, particularly at concentrations > 100 µM, suggesting that concentrations of nickel (II) below 100 µM do not interfere with copper (II) detection using the current approach. These findings demonstrate that the proposed method provides excellent selectivity for copper (II) detection even with the co-existence of various interfering metal ions.

### 3.3. Screening of Variables Affecting Copper (II) Detection

In this work, PBD was utilized to describe the association between three independent variables (CT, pH, and RM) and the dependent response (F_0_/F). The experimental data was fitted using the polynomial equation given in Equation (4).

The Box–Cox response transformation with a transformation factor, λ = −1, yielded the following regression equation:

(4) $$\frac{-F}{F_{0}}=-0.9377+0.03309\;\mathrm{pH}\;+0.001836\;\mathrm{Time}\;+0.02355\;\mathrm{RM}\;-0.04487\;\mathrm{Ct}\;\mathrm{Pt}$$

Ct Pt denotes the central point in the regression model defined by Equation (4). According to this model, all analyzed variables had a positive impact on the response, with pH having the highest effect. The suggested model’s reliability was further supported by the R^2^ (0.9544) and adjusted R^2^ (0.9485) values, which demonstrate a good correlation between experimental and model-predicted values. Also, the expected R^2^ was a higher value (0.9388), demonstrating the model’s ability for predicting every additional observation.

**Figure 7:**
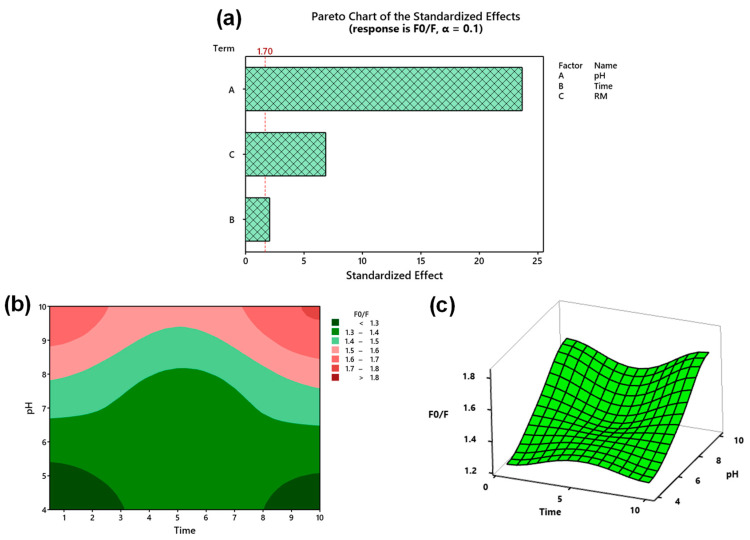
(**a**) Pareto chart of standardized effects, (**b**) 2D contour plots, and (**c**) 3D surface plots for pH and CT.

The Pareto chart, Figure 7a, depicts the relationship between the response, F_0_/F, and the three factors. As demonstrated, all variables exceed the reference line, indicating their statistical significance with pH (A) being the most statistically significant.

The 2D contour plots shown in Figure 7b were used to study the relationship between the response variable and two predictor variables, which are shown along the x- and y-axes. The x- and y-axes in Figure 7b indicate the two numerical variables, Time (C) and pH(A), respectively. The dark green contours represent an area where fluorescence quenching is lowest, with pH ranging from 4 to 5.4 and Time between ≈ 0 and 3 min and ≈ 8 and 10 min. The 3D surface plots were used to measure the relationship between the same two variables, but in a 3D format, with the *z*-axis representing the response variable. As illustrated in Figure 7c, the high ridges suggest that fluorescence quenching can be increased at both high pH (pH = 10) and Time (10 min). Analysis of variance (ANOVA) showed that all variables have a *p*-value < 0.05, confirming their statistical significance. The lack-of-fit showed a *p*-value that is greater than 0.05, implying insignificant lack-of-fit and hence good model linearity.

The individual desirability function (*d*) was used to determine the optimal experimental conditions that could simultaneously maximize the response variable. The desirability plot revealed that the highest fluorescence quenching could be reached by combining Time of 10 min, pH of 10, and using DIW as the RM. These best conditions were then used to generate the calibration curve and apply the standard addition approach.

The CDs’ surface charge could explain why the ideal pH was at a higher value. Under low pH conditions (acidic pH), the functional moieties such as carboxyl groups and other oxygenated moieties on the surface of CDs become protonated, resulting in a highly positively charged surface. This phenomenon causes the positively charged copper (II) to be repelled from the surface of the CDs. As a result, fluorescence quenching triggered by existence of copper (II) could be inefficient at low pH levels. When the pH rises, the functional groups on the surface of the CDs gradually deprotonate, allowing the interaction between the negatively charged surface and the copper (II) cations [65,66,67].

### 3.4. Method Validation

#### 3.4.1. Calibration Curve: Linear Range and Sensitivity

**Figure 8:**
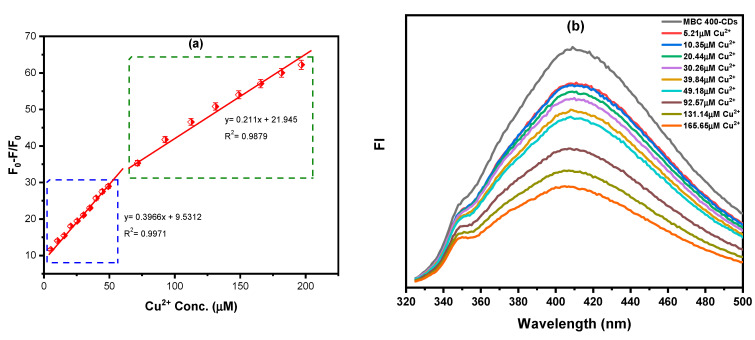
(**a**) The calibration curve for different concentrations of copper (II), determined using MBC400-CDs. (**b**) Fluorescence spectra of MBC400-CDs before and after adding different concentrations of copper (II).

The calibration curve obtained for detecting copper (II) using MBC400-CDs showed that as the concentration of copper (II) increased within the range of 4.9–56.6 µM and 56.6–197.5 μM, there was a consistent linear correlation between the concentration of copper (II) and the response, F_0_−F/F. This change was measured using excitation and emission wavelengths of 310 and 410 nm, respectively. Equations (5) and (6) represent this linear correlation.

(5) $$y=0.3966x+9.5312,\;\;R^{2}=0.9971$$

(6) $$y=0.211x+21.945,\;\;\;R^{2}=0.9879\;\;\;\;$$

where *y* indicates the response F_0_–F/F, and *x* is the concentration of copper (II). The high coefficient of determination, R^2^, suggests that the data exhibited strong linearity. Figure 8a shows the calibration curve for different concentrations of copper (II), determined using MBC400-CDs under the optimal experimental conditions. The fluorescence spectra of MBC400-CDs before and after adding different concentrations of copper (II) is shown in Figure 8b.

To assess the sensitivity of the proposed approach, the limit of detection (LOD) and limit of quantification (LOQ) were calculated operating the formulas in Equations (7) and (8), correspondingly, and using the values of the standard deviation (*σ*) of the blank sample, and the slope (*s*) of the linear calibration curve (first linear segment).

(7) $$LOD=3\times \frac{\sigma }{s}$$

(8) $$LOQ=10\times \frac{\sigma }{s}$$

For a linear range of 4.9–56.6 μM, the LOD is 0.01 µM, and the LOQ is 0.04 µM. These results highlight the remarkable sensitivity of the produced MBC400-CDs towards copper (II). The allowable level of copper in drinking water is 1.3 mg/L, which is approximately 20.5 µM. This value is significantly greater than our reported LOD (0.01 µM). In other words, the synthesized MBC400-CDs could detect copper (II) concentrations much below the allowable limit, making them useful candidates for environmental monitoring purposes. Furthermore, Table 7 shows a comparison of the performance of the mandarin peel-derived CDs with the other reported CD-based approaches for detecting copper (II). The comparison included both greenly [68,69,70,71,72,73] and chemically [74,75,76,77] synthesized CDs. As can be seen from Table 7, the mandarin peel biochar-derived CDs were working effectively over a wide concentration range. Moreover, comparing the performance of MBC400-CDs to the greenly synthesized CDs [68,69,70,71,72,73] revealed a reasonable LOD that is lower than the reported approaches. The LOD was also lower than the reported values using GQDs [76] and CDs derived from the pyrolysis of mixture of citric acid, sodium borohydride, and polyethyleneimine [77]. The NCDs (derived from urea and ethylenediaminetetraacetic acid) [74] and the CA-CdS QDs (obtained from citric acid, cadmium chloride, and thioacetamide) [75] showed a lower LOD; however, the procedure involved usage of hazardous chemicals at higher temperatures. The QY% is comparable to and sometimes higher than the reported CD-based sensors of green origin. Compared to the CDs from chemicals, the %QY of MBC400-CDs was lower; however, the current approach does not entail the use of any surface passivation, chemical modification, or doping. None of the reported approaches have employed a factorial design to control the variables affecting the sensing rehearsal.

#### 3.4.2. Accuracy and Precision

The accuracy and precision of the proposed procedure in the authentic samples were measured at a 95.0% confidence interval (95.0% CI), as shown in Table 8. The acquired data demonstrated a % mean recovery of 100.3 ± 3.2, validating the accuracy of the suggested approach. The % relative standard deviation was less than 5%, implying a high precision of the proposed procedure.

#### 3.4.3. Analysis of Real Samples

For real samples, the accuracy was tested using recovery studies after spiking the real samples with standard copper (II) solution, as shown in Table 9. Copper (II) recovery rates were 104 ± 0.85% in tap water compared to 102 ± 1.45% in the case of mountain water (Table 9), suggesting a good accuracy. These good results indicate that the matrix, which frequently contains other interfering components, has only a minor impact on the current sensor’s ability to detect copper (II).

### 3.5. Proposed Quenching Mechanism

Under UV irradiation at 310 nm, the CDs emit a strong fluorescence at 410 nm, resulting from electron excitation from the valence band (VB) to the conduction band (CB). With the addition of copper (II) to the CD solution, they interact with negatively charged functional groups on the surface of the CDs. This electrostatic interaction facilitates the splitting of the copper (II) *d* orbital energy levels, providing an alternative pathway for electron transfer. In this process, electrons from the excited state in the CB of the CDs transfer to the *d* orbitals of the copper (II) ions. The electrons were then returned to the ground state, bypassing the normal fluorescence pathway of the CDs. This electron transfer significantly reduces the fluorescence emission as the energy disruption alters the electronic transitions within the CDs, resulting in a quenching effect [78,79,80]. The degree of quenching is proportional to the concentration of copper (II) ions, making MBC400-CDs highly useful for detecting copper (II) in solutions with great sensitivity [77,81].

## 4. Conclusions

In conclusion, this study reports the green synthesis of carbon dots (CDs) derived from mandarin peel biochar (MBC) prepared at various pyrolysis temperatures. The CDs synthesized from MBC pyrolyzed at 400 °C (MBC400-CDs) exhibited remarkable luminescent properties, with a high quantum yield (QY) of 7.31%. The formed CDs at 400 °C showed small particles of 2.98 ± 0.50 nm, as confirmed by the TEM analysis. The MBC400-CDs showed strong emission intensity, with optimal excitation at 310 nm. After optimization of the experimental conditions affecting the sensing process using PBD, an ON–OFF copper (II) sensing system was established. The developed system showed a rapid response and reasonable selectivity with high sensitivity (a detection limit as low as 0.01 µM) over a wide linear response range (4.9–197.5 µM). Method validation confirmed the high accuracy and precision. The MBC400-CDs also showed exceptional performance in measuring copper (II) concentrations in real water samples, as reflected by the high precision and recoveries. The analytical technique developed in the current investigation has therefore demonstrated the potential of biochar-derived CDs for environmental applications.

## Figures and Tables

**Table 1:** The disadvantages of some precursors used for the synthesis of CDs.

Precursor	Drawbacks
Citric acid	One drawback is the complicated and poorly understood chemical mechanism that produces CDs from citric acid [24]. It can be challenging to regulate the optical characteristics of CDs made from citric acid, resulting in changes in their fluorescence and quantum yields [25]. Citric acid-synthesized CDs might also lack long-term photostability, which is crucial for bioimaging applications [26].
Glucose	Strong absorption in the observable range of wavelengths is a characteristic of glucose-based CDs that may restrict their use in some fields [27]. Also, producing CDs from glucose may involve complicated and time-consuming processes and toxic chemicals, leading to higher production costs [28].
Graphene	The high cost of synthesis and the possibility of contamination by strong acids are the major drawbacks of employing graphene as a carbon precursor [29,30].

**Table 2:** Factors and their corresponding levels provided to the PBD.

Code	Numerical Variables	−1	0	+1
A	Contact time, CT (min)	0.5	5.25	10
B	pH, (pH unit)	4	7	10
**Code**	**Categorical Variables**	**−1**		**+1**
C	Reaction medium, RM	DIW		ACN
**Code**	**Dependent Variables**			
Y	Fluorescence quenching, F_0_/F		Maximum	

**Table 3:** The PBD experimental matrix and the predicted fits (BPFITS).

Run Order	Pt Type	pH	Time	RM	F_0_/F	BPFITS
1	1	4	10	ACN	1.2600	1.2338
2	1	4	0.5	ACN	1.2300	1.2078
3	1	10	0.5	DIW	1.7180	1.7173
4	0	7	5.25	ACN	1.3131	1.3075
5	1	10	0.50	ACN	1.5378	1.5888
6	1	4	0.50	ACN	1.2260	1.2078
7	1	10	10	DIW	1.7470	1.7703
8	1	10	0.5	ACN	1.6116	1.5888
9	1	10	10	ACN	1.5906	1.6341
10	1	10	0.5	DIW	1.7180	1.7173
11	1	4	10	DIW	1.3620	1.3099
12	1	4	0.5	DIW	1.2670	1.2806
13	0	7	5.25	DIW	1.4070	1.3933
14	1	4	0.5	ACN	1.2450	1.2078
15	1	4	10	ACN	1.2530	1.2338
16	0	7	5.25	ACN	1.3668	1.3075
17	0	7	5.25	DIW	1.3889	1.3933
18	1	10	10	ACN	1.6460	1.6341
19	0	7	5.25	DIW	1.4137	1.3933
20	0	7	5.25	DIW	1.3979	1.3933
21	1	10	0.5	ACN	1.5384	1.5888
22	0	7	5.25	DIW	1.4171	1.3933
23	1	4	10	DIW	1.2601	1.3099
24	1	10	0.5	DIW	1.7987	1.7173
25	1	4	0.5	DIW	1.2617	1.2806
26	1	4	10	DIW	1.2486	1.3099
27	1	4	10	ACN	1.2390	1.2338
28	1	4	0.5	DIW	1.2391	1.2806
29	0	7	5.25	ACN	1.2948	1.3075
30	1	10	10	DIW	1.8120	1.7703
31	1	10	10	DIW	1.8232	1.7703
32	0	7	5.25	ACN	1.2437	1.3075
33	0	7	5.25	ACN	1.3370	1.3075
34	0	7	5.25	DIW	1.4058	1.3933
35	0	7	5.25	ACN	1.2384	1.3075
36	1	10	10	ACN	1.6240	1.6341

ACN: acetonitrile, DIW: deionized water, BPFITS: predicted fits.

**Table 4:** QY% of the obtained CDs compared to quinine sulfate.

Sample	Integrated Emission Intensity (I) Area	Refractive Index (η)	Absorbance (A) (at λ_ex_ = 314 nm)	QY (at λ_ex_ = 314 nm)
Quinine sulfate	8,051,819.586	1.33	0.052	
MBC400-CDs	$2.63\times 10^{6}$		0.127	7.31%
MBC600-CDs	$2.39\times 10^{6}$		0.149	5.66%
MBC800-CDs	$1.03\times 10^{5}$		0.03	1.21%

**Table 5:** A comparison between the biochar-sourced CDs and the CDs synthesized in the current approach.

Feedstock	Pyrolysis Temperature	Synthesis	%QY	Ref.
Avocado seeds	250, 400, and 600 °C	Hydrothermal synthesis	250 °C: 9.2% 400, 600 °C: ~2–3%	[46]
Purple moor grass biochar (*Molinia caerulea*) Spruce tree biochar (Picea) African oil palm biochar (*Elaeis guineensis*)	300–375 °C Not stated 200–400 °C	Hydrothermal synthesis in the presence of KMnO_4_	8.39% 5.44% 2.31%	[13]
Peanut shells	340–420 °C	Sonication	10.58%	[50]
Watermelon peels	Carbonization at low temperature	Sonication	7.1%	[51]
Mandarin peels	400, 600 and 800 °C	Hydrothermal synthesis	400 °C: 7.31% 600 °C: 5.66% 800 °C: 1.21%	This work

**Table 6:** Effect of different metal ions on MBC400-CDs–copper (II) fluorescence and the calculated tolerance limit.

Concentration of the Interferent Metal Ion (µM)	Tolerance Limit			
	Iron (III)	Nickel (II)	Chromium (III)	Cadmium (II)
5	1.05	1.26	0.21	0.17
10	1.43	2.69	0.38	0.33
20	2.19	3.88	0.72	0.59
40	2.79	6.38	1.32	1.19
60	3.48	8.22	1.59	1.51
80	4.03	9.03	1.72	1.68
100	4.27	10.07	2.17	1.94
150	5.79	13.13	2.63	2.51
200	7.41	16.08	3.14	3.04

**Table 7:** A comparison between different CD-based fluorescence sensors and the current approach for detecting copper (II).

Probe	Synthesis Method	Green Synthesis	Controlled Sensing	Linear Range	LOD	QY	Reference
BPEI-CQDs	Hydrothermal conversion of bamboo leaves at 200 °C followed by capping with branched polyethylenimine	Yes	No	1–140 μM	0.01 μM	7.1%	[68]
CQDs	Microwave-assisted carbonization of empty fruit bunch at 60–100 °C	Yes	No	0–400 μM	0.42 μM	Not stated	[69]
CDs_100–180_	Hydrothermal carbonization of biomass (hemicelluloses, lignin, chitosan, and a-cellulose) at 100–180 °C	Yes	No	0–30 μM	0.085 μM	2.8–16.6%	[70]
CNDs (nitrogen containing)	Hydrothermal treatment of pipe tobacco	Yes	No	0–40 μM	0.01 μM	3.2%	[71]
CQDs	Carbonization of petals of *Polianthes tuberose* L. at 300 °C for 8 h	Yes	No	0–70 μM	0.2 μM	3%	[72]
NCDs	Thermal coupling of lemon extracts and L-arginine at 200 °C for 3 h	Yes	No	0.05–300 μM	0.047 μM	7.7%	[73]
NCDs	Pyrolysis of urea and ethylenediaminetetraacetic acid at 200–300 °C for 1 h	No	No	0.001–22 μM	0.002 μM	11.26%	[74]
CA-CdS QDs	Hydrothermal synthesis from cadmium chloride and thioacetamide, using citric acid for surface modification	No	No	0.01–50 μM	0.009 μM	18.82%	[75]
GQDs	Hydrothermal method at 180 °C for 10 h from graphene oxide (reoxidized in mixture of concentrated sulfuric and nitric acids)	No	No	0–15 μM	0.226 μM	Not stated	[76]
CDs	Pyrolysis of mixture of citric acid, sodium borohydride, and polyethyleneimine at 180 °C for 1 h in an oil bath	No	No	0–80 μM	5.3 μM	25%	[77]
MBC400-CDs	Hydrothermal synthesis using renewable source, mandarin peels, at 180 °C for 4 h	Yes	Yes	4.9–56.6 μM	0.01 μM	7.31%	This work
				56.6–197.5 μM			

BPEI: branched polyethylenimine; CDs_100–180_: CDs prepared at temperatures from 100 to 180 °C, CNDs: Carbon nanodots; NCDs: nitrogen-doped carbon dots; CA-CdS QDs: citric acid-capped CdS quantum dots; GQDs: graphene quantum dots.

**Table 8:** Detection of copper (II) in authentic samples.

Taken (µM)	Found (µM)	% Recovery
5.21	5.27	101
10.4	10.8	104
15.4	14.9	96.8
20.4	21.4	105
25.4	25.0	98.4
30.3	29.1	96.0
35.1	34.0	96.9
39.8	40.8	103
44.5	45.4	102
49.2	48.9	99.4
Mean ± SD	100.3 ± 3.2 3.2	
% RSD		

**Table 9:** The concentration of copper (II) spiked in tap and mountain waters, as detected by the MBC400-CDs.

Sample	Copper (II)			
	Spiked (µM)	Found (µM)	Recovery%	RSD%
Tap water	0	2.43	-	0.78
	5.21	5.29	102	0.97
	10.4	11.1	107	1.28
	15.4	16.2	105	0.96
	20.4	21.3	104	0.68
	25.4	25.6	101	0.36
Mountain water	0	11.9	-	2.01
	5.21	5.34	102	1.29
	10.3	10.4	100	2.06
	15.4	15.7	102	2.01
	20.4	21.4	105	0.94
	25.4	25.6	101	0.36

## Data Availability

Data are contained within the article.
